# Enteropathogenic Bacterial and Intestinal Parasitic Infections among Asymptomatic Food Handlers in Rangsit University Canteens, Central Thailand

**DOI:** 10.1155/2021/5565014

**Published:** 2021-04-10

**Authors:** Sirima Kitvatanachai, Bajaree Jantrapanukorn, Utsanee Supcharoengoon, Chalirmporn Atasilp

**Affiliations:** ^1^Faculty of Medical Technology, Rangsit University, Pathumthani 12000, Thailand; ^2^Chulabhorn International college of Medicine, Thammasat University, Pathumthani 12121, Thailand

## Abstract

Food handlers play an important role in the transmission of foodborne diseases. 108 asymptomatic food handlers work in RSU canteens and have never been checked for intestinal bacteria and parasites, which might be a potential source of infection for customers. This study is aimed at estimating the prevalence of enteropathogenic bacterial and intestinal parasitic infections among food handlers in Rangsit University canteens, central Thailand. A total of 79 food handlers were enrolled, and each provided one stool sample (response rate of 73.2%). Females comprised 93.7% of study participants, and the largest age group was 41–50 years (34.2%). The prevalence of enteropathogenic bacteria in stool cultures was 2.5%, and only *Aeromonas* spp. were detected. The pathogenic protozoa *Giardia duodenalis* was detected in 1.3% of samples, and nonpathogenic protozoa was found in 11.4%. No helminths were found in any samples. Approximately 80% of food handlers demonstrated good hygiene practices, including regular hand washing after visiting the toilet, regular hand washing when preparing food, using soap when washing hands, wearing uniforms/gowns, practicing correct hand washing techniques, and having short fingernails. However, the results showed a lack of personal hygiene training and routine medical care (>50% of samples). Stronger intervention would help to eliminate future infections.

## 1. Introduction

Food handlers directly handle food or beverages or contact equipment used for food preparation. Asymptomatic food handlers play a significant role in food safety and are unaware of their potential to transmit foodborne diseases [1–3].

Foodborne diseases are a major cause of morbidity and mortality globally. Enteropathogenic bacteria, including *Salmonella* and *Shigella* and intestinal parasites, including *Tania saginata*, *Hymenolepis nana*, *Ascaris lumbricoides*, *Strongyloides stercoralis*, *Trichuris trichiura*, *Enterobius vermicularis*, and hookworms are examples; most prevalent intestinal protozoan diseases reported are *Giardia lamblia* and *Entamoeba histolytica* [4, 5]. About one-third of the world population is affected by foodborne disease annually causing two million deaths [6]. An estimated 1.9 million people in the world die annually from diarrheal disease; one-third of the population in developing countries is affected by microbial foodborne disease [7].

Prevalence rates of intestinal parasites in food handlers by wet mount method range from 3.7 to 52.2%. In separate studies, in Ethiopia, 14.8% [8], 41.1%, and 44.1% using direct mount and concentration technique of food handlers were infected; [9, 10], in Iran, 3.7% (wet mount method), 9% (wet mount, concentration technique, trichrome staining), 10.4% (wet mount, concentration technique; and 34.9% (wet mount, concentration technique, Ziehl-Neelsen and trichrome staining) of handlers infected [11–14]. Infection rates using direct mount and concentration technique were 15.5% in Mumbai [15] 3.7% in Jordan [16]. Using Lutz, modified Ritchie, and Ziehl-Neelsen techniques, it was 38.2% in Brazil [17]. Stool microscopic examination was 52.2% in Turkey [18].

Intestinal parasitic infections were assessed in food handlers in universities due to rapid growth of student population and campus facilities. Infection rate of parasitic infection using direct mount and concentration technique was 25.2% in Haramaya University cafeterias, eastern Ethiopia [19], 33.0% in Jimma University specialized hospital, southwest, Ethiopia [20], and 33.0% in Addis Ababa University, Ethiopia [21]. Parasitic infection rate of 10.3% by direct smear and Kato-Katz thick smear among food handlers in tourist area restaurants and educational-institution cafeterias in Sai-yok district, Kanchanaburi province, Thailand [22].

Enteropathogenic bacteria such as *Shigella boydii* was reported 0.9% of food handlers in Sari, Mazandaran province of Iran [23]. Mumbai, no *Shigella*, but *Salmonella typhi* isolated in 1% (2/200) of food handlers in a metropolitan tertiary care hospital [15]. Omdurman area of Sudan, one out of 259 food handlers infected with *Shigella boydii* and three with *Salmonella typhi* [24]. No enteric bacterial infections among food handlers in tourist area restaurants and educational-institution cafeterias in Sai-yok district, Kanchanaburi, Thailand [22], a tertiary care hospital, India [25], or hotels in the Dead Sea area, Jordan [16].

Gastrointestinal illnesses in Thailand, from Bamrasnaradura Infectious Diseases Institute revealed parasitic infections by wet smear, cultured in Jones' medium, modified acid-fast staining and Gram-chromotrope staining was 26.8%; bacterial infections by culture was 14.5%. The majority of intestinal parasitic and bacterial infections were of *Blastocystis* sp. (15.9%) and *Salmonella* spp. (8.7%) [26]. For the prevalence of helminthes and protozoan infections in patients, Thammasat University Hospital, Thailand, by concentration technique and wet smear, the top three highest helminthic infections are *Strongyloides stercoralis*, *Taenia* spp., and *Opisthorchis viverrini* with prevalence of 1.25, 0.37, and 0.15%, respectively. Protozoan infection, *Blastocystic* sp., *Entamoeba coli*, *E. histolytica*, and *Endolimax nana* were 0.02%, equally from 6,452 stool samples [27].

Thai Bureau of General Communicable Diseases Department reported 18.1% helminthiases (*Opisthorchis viverrini*, highest of 8.7%, hookworm infection 6.5%) and rotozoal infections 4.9% (*Sarcocystis hominis* the highest 1.5%) in Thailand using Kato thick smear and concentration method from 15,555 samples in 2008 [28].

In Pathum Thani province, Thailand, parasitic infections were 13.9–20.8% in school-aged children [29, 30]. Microbial and parasitic infections and personal hygiene habits of food handlers in this province are unknown. This study is aimed at assessing the incidence of enteropathogenic bacterial and intestinal parasitic infections and describing the personal hygiene habits of food handlers working in Rangsit University (RSU) canteens, since this asymptomatic group may serve as a source of infections and transmissions.

## 2. Materials and Methods

### 2.1. Study Design Area and Population

A cross-sectional study was conducted from May to July 2019 on the main campus of the Rangsit University (RSU) campus, located in the Lak-Hok subdistrict of Muang Pathum Thani District, Pathum Thani province, 28 km from the center of Bangkok and 16 km from Muang Pathum Thani. RSU canteens consist of 53 facilities, including kiosks, that serve food and drink and cater to 23,000 students (RSU registration information, 2019). There were 108 asymptomatic food handlers working during the period of this study.

### 2.2. Sample Data and Specimen Collection

The inclusion criteria include all food handlers who had direct contact with food and drink working in the Rangsit University student canteens and who provided informed consent were included. The exclusion criteria include food handlers who had diarrhea and fever, had taken antibiotics or antiparasitic drugs in the previous month or during data collection, and those who provided incomplete questionnaires were excluded from the study.

The sample size was determined using a single population proportion formula considering the following assumptions: *Z*_*a*_/2 = 1.96 for 95% level of confidence, level of precision = 5%, *P* = 0.20 [28]:

(1) $$\begin{array}{c} \frac{n={\left(z_{a}/2\right)}^{2}*P\left(1-P\right)}{d^{2}}=246. \end{array}$$

However, as the total number of the population was *N* = 108, a correction formula was used to adjust the sample size [21] as follows:

(2) $$\begin{array}{c} n=\frac{n}{1+\left(n/N\right)\;}=\frac{246}{1+\left(246/108\right)}=75. \end{array}$$

The minimum sample size was 75 food handlers. All food handlers were asked for participation by direct contact if they were working and met the criteria above for food safety inside the campus.

A structured questionnaire was used for face-to-face interviews to determine general demographic characteristics and information on type of work, age, sex, religion, ethnicity, educational level, responsibility, experience, and income. Personal hygienic status of each food handler on handwashing, fingernail trimming, and use of uniform and food sanitation training were included in questionnaire. A clinical sign of fever or diarrhea and if the person had taken antibiotics or antiparasitic drugs was asked as first screening.

One stool sample was collected from each participant in a clean stool cup. Stool macroscopic examination was observed and recorded before processing. All the stool samples were divided into two parts: one part for culturing within 2 hrs onto blood agar (Oxoid), MacConkey agar (Difco), and *Salmonella*-*Shigella* agar (Oxoid) plates. After 24 hr incubation at 37°C, the plates were examined. If there were suspected colonies, nonlactose fermenter (NLF) were taken and identified biochemically as the following: triple sugar iron agar, lysine iron agar, motility test, indole test, Simons citrate agar, urease test, ornithine decarboxylase test, malonate test, and mannitol fermentation test. Enteropathogenic bacteria were identified following standard procedures [31]. The remaining stool samples, 1-2 mg, was examined microscopically for intestinal parasites following wet mount preparation in normal saline (0.85% NaCl) and 1%iodine solution followed standard protocols [32, 33]. The modified formalin ether concentration was modified from the Ritchie technique of 1948 [34] to increase the potential of detecting intestinal parasites in the stool samples. About 2 g stool was filtered through two layers of wet gauze into a centrifuge tube. The volume was adjusted to 10 mL with 10% formalin and centrifuged at 600 g for 3 m. The supernatant was discarded, and 7 mL of 10% formalin and 3 mL of ether were added to the sediment and centrifuged at 600 g for 3 m. The supernatant was discarded, leaving only the sediment to which 3 mL of 10% formalin (as preservative) was added. The sediment was mixed before examination under a light microscope.

All samples were independently examined in a blinded fashion by two microscopists. Expert microbiologists and parasitologists reread all positive samples and 10% of randomly selected negative samples.

### 2.3. Data Analysis

Data were entered, checked for accuracy, and then analyzed descriptively (frequencies and percentages) using IBM SPSS software for Windows (Version 21.0).

Every individual with at least one positive test was considered positive for intestinal infections. The epidemiological of enteropathogenic bacterial and intestinal parasitic infections were reported on the percentage of prevalence and type of organism. Demographic data and personal hygienic status were analyzed and presented as frequencies.

### 2.4. Ethical Approval and Consent to Participate

Ethical clearance was obtained from the Ethical Review Committee, Rangsit University, Thailand (ethical clearance no. RSUERB2019-026). Written informed consent was obtained from study participants. Food handlers found to be positive for enteric pathogens and parasites were instructed to see a doctor at the hospital for treatment, depending on the type of species identified. All the participants were trained in proper handwashing techniques.

## 3. Results

### 3.1. Sociodemographic Data of RSU Food Handlers

A total of 79 food handlers (74 females (93.7%) and five males (6.3%)) out of 108 potential participants (73.2%) responded. The mean age of participants was 44.7 ± 13.9 SD. The largest food handler age group was 41–50 years (34.2%). The vast majority were Buddhist (95.0%), 92.4% were Thai nationals, 65.8% were formally educated, and 26.6% had no education. The largest food handler group was sellers (38.0%). More than half of the participants had 1–5 years of work experience, and more than half were middle income, earning 9,001–16,000 Baht (USS 300-533)/month (Table 1).

### 3.2. The Prevalence of Enteropathogenic Bacterial and Intestinal Parasitic Infections in RSU Food Handlers

Out of 79 stool cultures tested for enteropathogenic bacteria, two samples (2.5%) were positive for *Aeromonas* spp. Combining wet mount and the modified formalin-ether concentration methods for parasitic intestinal infections, 9 (11.4%) out of 79 samples were found to be positive for at least one or more parasite species. Of these, 8 (10.1%) were infected with nonpathogenic protozoa and one (1.3%) with the pathogenic protozoa of *Giardia duodenalis*. No helminths were detected in any samples from RSU food handlers (Table 2).

### 3.3. Personal Hygiene among Food Handlers

Of the total respondents, 64 (81.0%) used the correct handwashing method, 77 (97.5%) reported that they regularly washed their hands when preparing food, 76 (96.2%) used soap during handwashing, all regularly washed their hands after visiting the toilet, 63 (79.8%) had fingernail trimming, 76 (96.2%) used uniforms/gowns when working, 50 (63.3%) wore a cap, 42 (53.2%) had passed food sanitation training, 28 (35.4%) had food sanitation training certificates, 13 (16.5%) had food handling certificates, and 48 (60.8%) had medical checkups (Table 3).

### 3.4. Identification of Isolated Pathogenic Organisms

The two food handlers infected with *Aeromonas* spp. were females, ages 53 and 58, were formally educated. One identified as an owner (a person who owns the shop and organizes everything in the shop, such as presentation of food and calculating the price of food/drink) and one a seller (a person who hands the food or drink to customer), and both performed regular hand washing when preparing food. However, one of them did not use soap when washing hands, one had long fingernails, one had never had a medical checkup, one had never passed food sanitation training, one had no food sanitation-training certificate, and one had no food handling certificate.

*Giardia duodenalis* was detected in one female, age 42 years, from Laos, with no education. She identified as a cook, but she did not know proper handwashing techniques, did not have regular medical checkups, had never passed food sanitation training, and did not have certificates for either food sanitation training or food handling.

## 4. Discussion

Among food handlers in Rangsit University canteens, the prevalence of enteropathogenic bacteria was 2.5%. Despite a parasitic intestinal infection rate of 11.4%, there was only one pathogenic protozoan infection (*Giardia duodenalis*), while 10.1% were nonpathogenic protozoan infections. However, this study was performed using one stool sample due to the limited time of food handlers to provide the stool sample and it might be better if we could have collected 3 stool samples to reach the true prevalence. Although available data on enteropathogenic bacterial and parasitic infection are limited in some area of Thailand and out of date, Kusolsuk et al. (2011) found parasitic infections in 10.3% (and no enteric bacterial infections) of food handlers in tourist area restaurants and school cafeterias in the Sai-yok district, Kanchanaburi province, Thailand. Hookworms represented the greatest number of parasitic infections (7.7%) [22], in contrast to this study, in which there were no helminthic infections. In three suburban government schools in the Lak Hok subdistrict, Mueang Pathum Thani, Thailand, about 4-6 kms away from Rangsit University. It was reported that 13.9% of samples were positive for intestinal parasites, and just one case presented hookworm infection using the same methods of this study [29]. However, in 2018, the study in school children in the same area also showed no helminthic infections similar to our study [30]. This may be the suburbanization effect on people to have better hygiene, to use toilet, to eat cooked food, to wear shoes for work, and also less number of people working in agriculture. The highest protozoan infection was with *Giardia duodenalis*, which is consistent with this study. In the same subdistrict in 2018, the prevalence of parasitic intestinal infection in one school was 20.8% (5.2% pathogenic parasites and 15.6% nonpathogenic parasites). *Giardia duodenalis* (2.1%) and *Blastocystis* sp. (>5 cells/HPF) (2.1%) were the most dominant species of pathogenic protozoa. *Blastocystis* sp. (<5 cells/HPF) were the most prevalent (11.4%) [30]. This species was also found in this study, although the prevalence was lower (5.1%). It may be that protozoa are more prevalent in children than adults because they pay less attention to hygiene.

*Aeromonas* spp. were found in two samples (2.5%), which is consistent with other studies that found no enterobacterial infection from stool cultures among food handlers in Kanchanaburi province of Thailand [22], in a tertiary care hospital of India [25], or the Dead Sea area of Jordan [16]. Enterobacteria in stool cultures have been reported from other areas: *Shigella boydii* was found 0.9% in northern Iran and 1.3% in the Omdurman area of Sudan [23, 24], *Samonella typhi* was found in 1% of samples collected in a tertiary care hospital of Mumbai and 1.3% in the Omdurman area of Sudan [15, 24], and *Salmonella* was isolated from 3.5% of samples in Addis Ababa, Ethiopia [21].

*Aeromonas* spp. was the only enterobacteria found in food handlers in RSU canteens. Identification to the species level using phenotypical characterization is difficult due to the variable of the strains, which can cause a lot of confusion. The genus *Aeromonas* is diverse (at least 16 DNA hybridization groups) [35]. *Aeromonas hydrophila*, *A. caviae*, and *A. veronii* biotype sobria are considered clinically significant [36]. Identification of *Aeromonas* to species level requires genetic techniques not utilized in this study. *Aeromonas* species can cause gastroenteritis, septicemia, and extraintestinal, wound, urinary tract, hepatobiliary, and ear infections. However, *Aeromonas* spp. can cause diarrhea in healthy carriers [37].

Food handlers at RSU canteens showed good personal hygiene (≥80%), i.e., regular hand washing after visiting toilets, regular hand washing when preparing food, using soap when washing hands, using uniforms/gowns, performing the correct hand washing method, and having short fingernails. Notably, <50% of the food handlers had passed food sanitation training and had a medical checkup, which may be because many food handlers work for a short period and then leave. However, personal hygiene and food safety interventions still need to improve to reach acceptable hygiene standards for customer safety. To this end, our team arranged for the food handlers to be trained in correct hand washing techniques.

This study has some limitations: (a) Because of low sensitivity of conventional microscopy, the reported prevalence rates for enteric parasites may represent an underestimation of the true figures. This is enhanced by the fact that a single stool per participant was analyzed; however, the modified formalin ether concentration were performed to support the prevalence. (b) No specific techniques were used for certain parasitic pathogens including coccidian (e.g., Ziehl-Neelsed staining) and soil-transmitted helminths such as *Strongyloides* (e.g., Baermann method), so it is likely that some parasitic species were undetected. (c) No molecular methods were used for genotyping purposes (e.g., for *G. duodenalis* and *Blastocystis* sp.). This would add important information about the species and genetic variants circulating in the investigated human population. This information would be extremely useful to ascertain sources of infection and transmission pathways.

## 5. Conclusion

Though we detected a low prevalence of enteropathogenic bacteria (2.5% of *Aeromonas* spp.) and pathogenic protozoa (1.3% of *Giardia duodenalis*) in RSU food handlers, to reach the criteria of food sanitation and food safety, food handlers working in RSU canteens must participate in food sanitation training and have medical checkups annually to certify the shop safe enough to sell food and drink. The administrator should cooperate with the shops to support all staff in reaching hygiene goals.

## Figures and Tables

**Table 1:** Sociodemographic data of food handlers in RSU student canteens (*n* = 79).

Demographic characteristics	Frequency	% of total
Types		
Beverage	15	19.0
Food	48	60.8
Staff	16	20.3
Sex		
Male	5	6.3
Female	74	93.7
Age (years)		
≤20	1	1.3
21–30	12	15.2
31–40	13	16.5
41–50	27	34.2
50–60	15	19.0
>60	11	13.9
Religion		
Buddhism	75	94.9
Islam	4	5.1
Ethnicity		
Thai	73	92.4
Laos	2	2.5
Burmese	3	3.8
Cambodian	1	1.3
Education		
Illiterate	21	26.6
Primary and secondary school	52	65.8
No answer	6	7.6
Responsibility		
Owner	18	22.8
Cook	18	22.8
Seller	30	38.0
Owner and seller	9	11.4
Cook and seller	3	3.8
Prepare food/wash dishes and clean	1	1.3
Experience (years)		
<1	6	7.6
1-5	43	54.4
6-10	11	13.9
>10	16	20.3
No answer	3	3.8
Incomes (Baht)		
<9,000	26	32.9
9,001-16,000	42	53.2
16,000-30,000	6	7.6
>30,000	5	6.3

**Table 2:** Incidence of enteropathogenic bacterial and intestinal parasitic infections from the stools of beverage and food handlers at RSU canteens (*n* = 79).

Types of intestinal organisms	Frequency	% of total
Enteropathogenic bacteria		
*Aeromonas* spp.	2	2.5
Intestinal parasites	9	11.4
Nonpathogenic protozoa	8	10.1
*Endolimax nana*	2	2.5
*Blastocystis* sp. (<5 cells/HPF)	2	2.5
*Endolimax nana Blastocystis* sp.	1	1.3
*Endolimax nana+*		
*Entamoeba histolytica*/*dispar*	2	2.5
*Endolimax nana+Blastocystis* sp.+		
*Entamoeba histolytica*/*dispar*	1	1.3
Pathogenic protozoa	1	1.3
*Giardia duodenalis*	1	1.3
Total	11	11.9

**Table 3:** Personal hygiene among beverage and food handlers at RSU canteens (*n* = 79).

Personal hygiene practice	Yes (%)	No (%)
Correct handwashing method	64 (81.0)	15 (19.0)
Regular hand washing when preparing food	77 (97.5)	2 (2.5)
Use of soap when washing hands	76 (96.2)	3 (3.8)
Regular hand washing after visiting toilet	79 (100.0)	0 (0.0)
Fingernail trimming	63 (79.8)	16 (20.3)
Use of uniform/gown	76 (96.2)	3 (3.8)
Wearing cap	50 (63.3)	29 (36.7)
Food sanitation training	42 (53.2)	37 (46.8)
Having certificate of food sanitation training	28 (35.4)	51 (64.6)
Having certificate of food handlers	13 (16.5)	66 (83.5)
Frequency of medical checkup		31 (39.3)
Every 3-6 months	11 (13.9)	
Every 6-12 months	35 (44.3)	
Others	2 (2.5)	

## Data Availability

The data used to support the findings of this study are included within the article.
